# The validity of animal models to explore the pathogenic role of the complement system in multiple sclerosis: A review

**DOI:** 10.3389/fnmol.2022.1017484

**Published:** 2022-10-13

**Authors:** Nil Saez-Calveras, Amy L. Brewster, Olaf Stuve

**Affiliations:** ^1^Department of Neurology, University of Texas Southwestern Medical Center, Dallas, TX, United States; ^2^Neurology Section, Parkland Hospital, Dallas, TX, United States; ^3^Department of Biological Sciences, Southern Methodist University, Dallas, TX, United States; ^4^Neurology Section, VA North Texas Health Care System, Dallas, TX, United States; ^5^Peter O’Donnell Brain Institute, University of Texas Southwestern Medical Center, Dallas, TX, United States

**Keywords:** complement system, multiple sclerosis, experimental autoimmune encephalomyelitis, progressive multiple sclerosis, synaptic pruning, adaptive immune response, animal models, opsonization

## Abstract

Animal models of multiple sclerosis (MS) have been extensively used to characterize the disease mechanisms in MS, as well as to identify potential pharmacologic targets for this condition. In recent years, the immune complement system has gained increased attention as an important effector in the pathogenesis of MS. Evidence from histological, serum, and CSF studies of patients supports an involvement of complement in both relapsing-remitting and progressive MS. In this review, we discuss the history and advances made on the use of MS animal models to profile the effects of the complement system in this condition. The first studies that explored the complement system in the context of MS used cobra venom factor (CVF) as a complement depleting agent in experimental autoimmune encephalomyelitis (EAE) Lewis rats. Since then, multiple mice and rat models of MS have revealed a role of C3 and the alternative complement cascade in the opsonization and phagocytosis of myelin by microglia and myeloid cells. Studies using viral vectors, genetic knockouts and pharmacologic complement inhibitors have also shown an effect of complement in synaptic loss. Antibody-mediated EAE models have revealed an involvement of the C1 complex and the classical complement as an effector of the humoral response in this disease. C1q itself may also be involved in modulating microglia activation and oligodendrocyte differentiation in these animals. In addition, animal and *in vitro* models have revealed that multiple complement factors may act as modulators of both the innate and adaptive immune responses. Finally, evidence gathered from mice models suggests that the membrane attack complex (MAC) may even exert protective roles in the chronic stages of EAE. Overall, this review summarizes the importance of MS animal models to better characterize the role of the complement system and guide future therapeutic approaches in this condition.

## Introduction: Animal models of multiple sclerosis and the complement system

Multiple sclerosis (MS) is a chronic inflammatory disease of the central nervous system (CNS) characterized by extensive demyelination of the white matter, as well as axonal and neuronal loss. It represents the most prevalent cause of non-traumatic disability among the young adult population and is estimated to affect almost 1 million people in the US (Ghasemi et al., 2017; Wallin et al., 2019). This disease can follow three main clinical presentations: A relapsing-remitting (RRMS) phenotype, with exacerbations and periods of clinical stability, a secondary progressive (SPMS), and a primary progressive course (PPMS) (Noseworthy et al., 2000). Although therapeutic options exist for the management of RRMS, treatments for progressive MS are currently scarce and represent an area of extensive investigation (Faissner and Gold, 2019). The pathogenesis of MS involves complex interactions between the adaptive immune system, the innate immune system, and neural and glial cells within the CNS. However, a specific trigger for the pathogenesis of MS remains to be fully elucidated. In addition, the mediators of these interplay between the innate and the adaptive immune response also remain to be better characterized. With the aim of understanding the mechanisms of disease initiation and progression in MS, as well as to identify potential clinical targets for this condition, the use of animal models is of outmost importance.

The most commonly used animal model of MS is the mouse model of experimental autoimmune encephalomyelitis (EAE), where autoimmunity to CNS components is induced through immunization with self-antigens derived from myelin (Smith, 2021). Complete Freund’s adjuvant (CFA), a water in oil emulsion with inactivated mycobacteria, and pertussis toxin (PT) are added as co-adjuvants of the antigens to facilitate the induction of EAE. These adjuvants can also induce the oscillatory pattern of relapsing-remitting disease in some mouse strains (Zamvil and Steinman, 1990; Baxter, 2007). EAE may be induced in mice through either active immunization with a protein or peptide (active EAE) or passive transfer of encephalitogenic T cells (transferred or passive EAE). The relevant immunogenic proteins include myelin basic protein (MBP), proteolipid protein (PLP), and myelin oligodendrocyte glycoprotein (MOG). Immunization of SJL/J mice with the immunodominant epitope of PLP (PLP_139–151_) induces a relapsing-remitting disease course (Tuohy et al., 1989), while disease induced by the immunodominant MOG_35–55_ peptide in C57BL/6 mice leads to a persistent phenotype (Tompkins et al., 2002). Immunization of Biozzi ABH mice with spinal cord homogenate (SCH) can also lead to relapsing-remitting disease that can progress to a more persistent course as these mice age (Peferoen et al., 2016). Beyond mice, other animal species including guinea pigs, rats, and monkeys have been used. However, only mice (Olitsky and Yager, 1949) and rats (Lipton and Freund, 1952) resulted in the best animal models to evaluate acute monophasic, relapsing-remitting, and persistent disease. After induction, EAE pathogenesis is typically characterized by the migration of activated myelin-specific T cells to the CNS through the blood brain barrier (BBB). The synthesis of chemokines and cytokines by these T cells also attracts an influx of monocytes and phagocytes to the CNS lesions (Miller and Karpus, 2007). The activation of these peripheral cells as well as CNS resident microglia leads to the formation of demyelinating and inflammatory lesions (Kurschus, 2015). Therefore, EAE recapitulates multiple features of human MS and represents a relevant model for this condition.

In addition to EAE, other animal models have also been developed to interrogate the underlying causes of MS. The chemically induced models of demyelination are based on the administration of molecules that specifically target oligodendrocytes, the myelinating cells of the CNS, causing their degeneration and death (Rodriguez, 2007). Among these, cuprizone is a neurotoxic copper-chelator agent that represents the most commonly used compound for these intoxication models of MS (Praet et al., 2014). This agent can lead to oligodendrocyte apoptosis, axonal pathology, glia activation and immune cell infiltrates into the brain (Ludwin, 1978; Hiremath et al., 1998). The disease is usually induced in C57BL/6 mice where, depending on the pattern of cuprizone administration, these animals can recapitulate the course of acute, relapsing-remitting, or chronic disease seen in human MS (Palumbo and Pellegrini, 2017). Epidemiological studies and molecular studies have linked viral infections (i.e., EBV, HVV6) to the pathogenesis of MS (Stuve et al., 2004; Virtanen et al., 2011; Tzartos et al., 2012; Mecha et al., 2013; Meier et al., 2021; Bjornevik et al., 2022). To further explore this, viral models of chronic demyelination in animals have also been developed. The Theiler’s murine encephalomyelitis virus (TMEV)-induced demyelinating disease model leads to a chronic progressive disease phenotype (Friedmann and Lorch, 1985; Mecha et al., 2013). Intracerebral infection of SLJ/J mice with this virus causes immune cell infiltration into the CNS and epitope spreading from viral to self-myelin antigens, leading to chronic demyelinating injury (Katz-Levy et al., 1999). This model recapitulates some of the features of progressive MS, including progressive loss of memory and sensory functions (Gilli et al., 2016b), compartmentalized inflammation and predominance of innate immune responses in the late stages (Gilli et al., 2016a). A different mouse model employs the murine herpesvirus (MHV). This virus infects and replicates in oligodendrocytes leading to demyelination both through a direct cytotoxic effect of the virus and through immune-mediated mechanisms (Lavi et al., 1984; Lane and Hosking, 2010). Other viral MS models also include the Semliki Forest Virus (SFV) (Fazakerley and Webb, 1987) and Sindbis Virus (SV) models (Metcalf and Griffin, 2011).

Overall, given their ability to replicate some of the pathogenesis and different phenotypes of MS, animal models represent a good template to explore the pathophysiology mechanisms of this condition.

**FIGURE 1:**
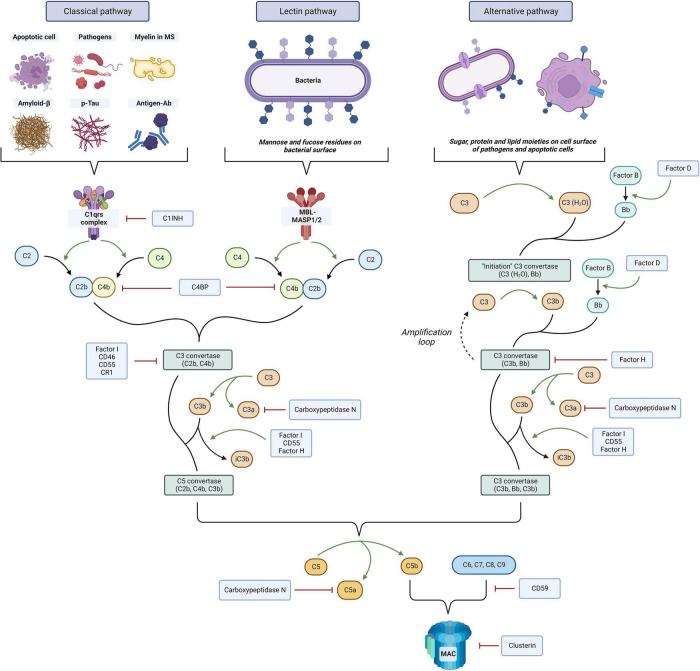
Overview of the immune complement system. Three distinct pathways can be activated, the classical, lectin, and alternative. Complement activation ultimately leads to cell lysis through membrane attack complex (MAC) formation on the cell surface, antigen opsonization and phagocytosis by C3 fragments, and proinflammatory and chemotactic effects mediated by chemokines C3a and C5a. The system is tightly regulated at multiple levels by complement regulators that prevent its overactivation. The figure was created with BioRender.com.

In recent years, the immune complement system has gained increased attention as a critical effector of the innate immune response in numerous diseases. The complement system consists of more than 30 distinct plasma proteins that interact with each other and act as critical effectors of the innate immune through the targeting and clearance of pathogens as well as dead and apoptotic cells (Sarma and Ward, 2011). Figure 1 offers an overview of the complement cascade. Complement proteins have also been identified as key mediators of the interaction between the innate and adaptive immune system. In the CNS, this system is presumed to participate in the pathogenesis of multiple autoimmune and neurodegenerative conditions, including Alzheimer’s disease (AD) (Morgan, 2018), stroke, traumatic brain injury, spinal cord injury, and amyotrophic lateral sclerosis (ALS) (Carpanini et al., 2019). In line with these findings, multiple evidence also appears to link the immune complement system with the pathogenesis and disease mechanisms of MS. As it will be described below, in histological studies complement proteins were found to be consistently positive in white matter plaques and the gray matter (Michailidou et al., 2015) of patients with RRMS (Breij et al., 2008) and progressive MS (Ingram et al., 2014). Serum and CSF studies of patients with MS also showed changes in the levels of these complement factors (Ingram et al., 2010a,b; Aeinehband et al., 2015; Tatomir et al., 2017).

Animal models of MS have also been extensively used to evaluate the effects of complement modulation in this disease. Therefore, in this review, we aimed to explore the multiple pharmacogenetic approaches of complement modulation that have been trialed in animal models of MS. These models can serve as a platform to better characterize the role of the complement system in this condition and help develop new therapeutic interventions.

## Complement in multiple sclerosis: An overview of clinical, histopathological, and serological evidence

As reviewed extensively by Ingram et al. (2009) the immune complement system may play an important role in MS, as well as in multiple other neuroinflammatory and neurodegenerative conditions (Dalakas et al., 2020; Schartz and Tenner, 2020). Here we wanted to provide a brief overview of the clinical evidence for the involvement of complement in human MS. Complement activation in MS white matter plaques has been extensively described in immunohistochemical studies (IHC) of *post-mortem* MS brains. MS plaques have been found to be consistently positive for complement proteins, activation products, complement receptors, and regulators in active plaque and peri-plaque areas, as well as in normal appearing white matter (NAWM) and cortical regions (Schwab and McGeer, 2002; Ingram et al., 2014). These findings have been observed both in RRMS and progressive MS cases (Prineas et al., 2001; Ingram et al., 2014; Watkins et al., 2016; Morgan et al., 2020). In NAWM and periplaque areas of chronic MS patients, complement activation may even occur independently of immune cell-mediated demyelination (Morgan et al., 2020). Isolated microglial nodules containing short, linear deposits of activated complement (C3d) overlying partly demyelinated axons have been described in these regions and may act to remove damaged axons (Barnett et al., 2009). Similarly, C1q and C3d deposition has been observed in the CA2/CA3 hippocampal regions of MS brains (Michailidou et al., 2015). These components may be involved in microglia-mediated synaptic loss in a process that could be dependent on intrinsic neuronal cell death signaling (Bode et al., 2014; Erturk et al., 2014; Gyorffy et al., 2018; Morgan et al., 2020).

Further evidence for the role of complement in MS comes from serological studies. Increased plasma levels of C3, C4, C4a, C1 inhibitor, factor H and reduced levels of C9 were also seen in MS patients compared to controls (Ingram et al., 2012). In the CSF, C3, and C4b levels were found to be elevated (Li et al., 2011) while factor H, was decreased in active MS but increased in progressive disease (Ingram et al., 2011). Factor H levels were also found to be an effective indicator of disease progression and served as a biomarker to stratify the disease course (Ingram et al., 2010a). In a different study, plasma C4a was raised during acute relapses of MS, while CSF C4a remained significantly elevated throughout the disease course in MS patients (Ingram et al., 2010b). This clinical evidence suggests that complement synthesis, activation and regulation may be important for the course of this disease.

Despite this, to this day, no clinical studies have been conducted exploring the inhibition or modulation of complement activity in MS. The only currently approved complement inhibitor in the context of demyelinating disease is eculizumab, a C5 inhibitor antibody, that was FDA-approved for use in neuromyelitis optica spectrum disorder (NMOSD) (Frampton, 2020). Therefore, the use of complement knockout (KO) models and pharmacological complement inhibitors in animal models of MS can help elucidate the role of immune complement signaling in this disease and advance the development of complement therapeutics to control the burden of MS. In the following sections we will review the pharmacogenetic approaches developed to this date that explored this relationship.

## Understanding the role of early complement components and its modulation on the course of experimental autoimmune encephalomyelitis

### Complement C3 and the alternative complement cascade

**FIGURE 2:**
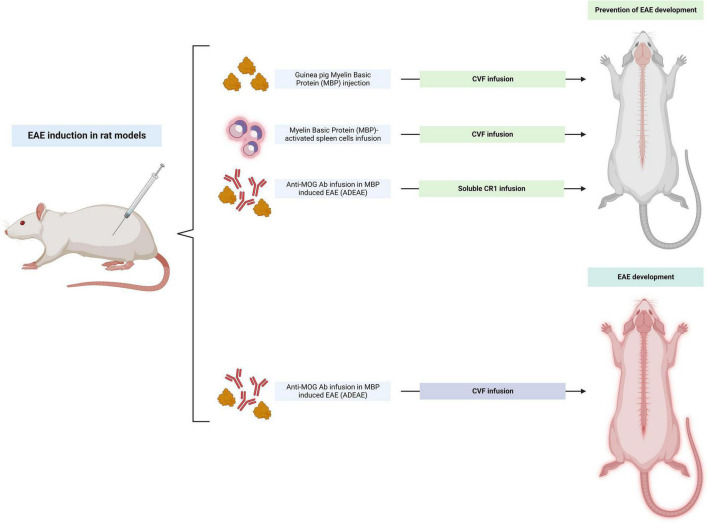
Summary of studies on the modulation of “upstream” complement factors C1 and C3 in rat models of experimental autoimmune encephalomyelitis (EAE). Cobra venom factor (CVF) infusion improved the disease phenotype in myelin basic protein (MBP) injected rats and in rats infused with MBP. While soluble CR1 infusion was able to prevent disease in anti-myelin oligodendrocyte glycoprotein (MOG) mediated ADEAE, CVF infusion was not. The figure was created with BioRender.com.

Complement activation is not only evident in clinical studies but also in experimental models of MS. After EAE was induced in male Lewis rats by injection of MBP together with CFA, complement C3 was found to be significantly elevated at day 10 and day 14 after immunization (Rosenling et al., 2012). In marmoset monkeys, MOG-induced EAE was capable of causing MS pattern II lesions that exhibited antibody and complement colocalization and damage (Merkler et al., 2006b). The first studies that evaluated the modulation of complement activity in animal models used the synergistic model of acute EAE in the Lewis rat. In these rats, transient depletion of complement C3 and C5 with cobra venom factor (CVF) suppressed the clinical expression of acute inflammatory EAE induced by immunization with guinea pig basic MBP, or by passive transfer of MBP-activated spleen cells (Abrahamson, 1971; Pabst et al., 1971; Linington et al., 1989b; Figure 2). In early disease, CVF also decreased the severity of CNS neuroinflammation although this difference disappeared with time in CVF-treated *vs.* wild-type (WT) EAE rats. However, the disease phenotype remained more severe in the latter. In addition, treatment with CVF reduced the demyelinating effect of a systemic injection of the anti-MOG monoclonal antibody which can lyse oligodendrocytes in a dose and complement-dependent manner. Despite this initial success, CVF injections appear to have only a transient effect on complement depletion. In addition, although CVF treatment in MBP-induced EAE rats causes suppression of disease, in antibody-mediated EAE models CVF administration had no effect on the disease course despite preventing C9 deposition (Piddlesden et al., 1991). Beyond CVF, treatment with soluble C receptor 1 (sCR1), which blocks C3 and C5 convertase activity, was capable of inhibiting inflammation and demyelination in antibody-mediated EAE in rat models (Piddlesden et al., 1994). However, this treatment was not as effective as CVF in rescuing the EAE disease course in these animals (Vriesendorp et al., 1997; Figure 2).

**FIGURE 3:**
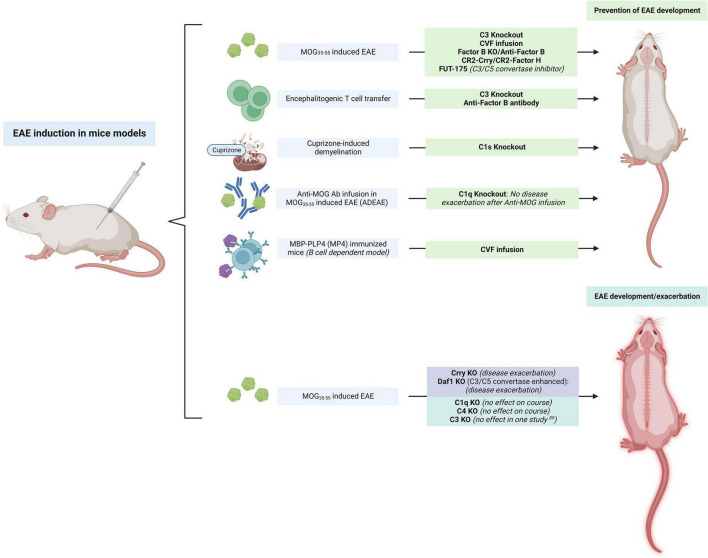
Summary of studies on the modulation of “upstream” complement in mice models of experimental autoimmune encephalomyelitis (EAE). The inhibition of C3 had largely beneficial effects in preventing the development of EAE, while the inhibition of complement regulators (Crry, Daf1) exacerbated the disease. C1q KO had no effect on the disease course but prevented phenotype worsening after anti-myelin oligodendrocyte glycoprotein (MOG) antibody infusion. The figure was created with BioRender.com.

The initial evidence for the effects of complement inhibition in rats was translated to mouse models (Figure 3). When transient complement depletion was induced by a single injection of CVF 2 days before induction of disease in MOG_35–55_-induced EAE C57BL/6 mice, the onset of disease was significantly delayed. In these mice the level of MOG-specific autoantibodies and their complement activating capacity at 3°weeks was significantly reduced (Terenyi et al., 2009). The proliferative capacity of MOG-specific T lymphocytes derived from these CVF treated animals was also decreased, and their spinal cords showed lower infiltrates of CD4 + T cells. Similar results after CVF treatment were observed when EAE was induced with PLP_139–151_ peptide injection in SJL/J mice. These animals also had attenuated relapsing remitting EAE (Terenyi et al., 2009).

MOG_35–55_-induced C3 KO EAE mice developed a significantly less severe disease phenotype as assessed by the EAE clinical score when compared to WT mice (Figure 3). Interestingly, C3 ± mice (which had a 50% reduction in C3 levels in serum) had an intermediate phenotype between that of WT and C3 KO mice (Szalai et al., 2007). The transfer of encephalitogenic T cells to C3 KO mice also resulted in an attenuated EAE profile. These mice had fewer CD4 + and CD8 + T cells in the CNS and 50% less of these cells produced IFNy when compared to the WT mice. In another study, C3 KO and factor B KO mice immunized with MOG_35–55_ to induce EAE also had a marked reduction in disease severity (Figure 3). Although EAE induction in these mice led to meningeal and perivascular inflammation, C3 KO and factor B KO mice had little infiltration of the parenchyma by macrophages and T cells, and these animals were also protected from demyelination (Nataf et al., 2000). When Hu et al. treated MOG_35–55_-induced EAE mice with administration of an anti-factor B antibody prior to the onset of clinical signs of EAE, no effects were seen in the onset and severity of the acute phase of disease. However, a significant attenuation of the chronic phase of the disease was observed in these animals resulting in reduced cellular infiltration, inflammation, and demyelination (Hu et al., 2013; Figure 3). This attenuation of the chronic phase of the disease was long-lasting even though administration of the antibody was terminated shortly after onset of disease with no further infusions after. Interestingly, transferred EAE mice with MOG-specific encephalitogenic T cells also had an attenuated disease course when anti-factor B antibody was administered before or after disease onset. This evidence supports a potential benefit of anti-factor B therapies in chronic demyelinating disease, where therapeutic options are limited.

In a different study, targeted inhibition of complement using complement receptor 2 (CR2) conjugated inhibitors significantly attenuated EAE (Figure 3). Treatment with CR2-Crry, a C3 inhibitor, and CR2-factor H which inhibits the alternative pathway, had protective effects in MOG_35–55_-induced EAE. Both inhibitors were capable of delaying the onset of EAE and these animals also had attenuated disease as assessed by their clinical score once the disease developed (Hu et al., 2012). Interestingly, deletion of the complement C3 inhibitor Crry in mice induced microglial priming as assessed by CD11b + microglial hypercellularity (Ramaglia et al., 2012). These microglia were highly ramified and had an activated morphology, and a LPS challenge overactivated these primed microglia to produce proinflammatory molecules (IL-1β, TNF). On neuropathological exam, significant colocalization of C3b/iC3b and CR3 was seen in the spinal cord of these mice. In contrast, mice that were double KO for C3 and factor B did not show this microglial priming (Ramaglia et al., 2012). When EAE was induced in these mice, the disease was significantly exacerbated in those that were Crry deficient. This suggests that a C3-dependent uncontrolled microglial priming may confer susceptibility to inflammatory challenges. In another study, astrocyte targeted production of soluble Crry, fully protected or delayed the development of clinical signs in EAE mice (Davoust et al., 1999b).

These results indicate the importance of complement activation, and especially of the alternative complement pathway, in the pathogenesis of disease. This also shows that complement-mediated events occur early during the effector phase of EAE and its effects extend into the chronic stages of this condition (Davoust et al., 1999b). To provide further evidence for the predominant involvement of the alternative complement in MOG-induced EAE models, C1q KO did not prevent MOG-induced EAE in C57BL/6 mice (Urich et al., 2006). Similarly, C4 deficient MOG_35–55_-induced EAE mice were found to have an onset and progression that was virtually identical to WT mice (Morariu and Dalmasso, 1978). Deletion of the C4 gene did not significantly change either the time of onset or the severity and tempo of MOG-induced EAE compared with controls. Similar levels of cellular infiltration by CD11b + macrophages and CD3 + T cells, and demyelination were also seen (Boos L. A. et al., 2005).

Recent evidence from experimental animal models also suggests that dimethyl fumarate (DMS), a well-known agent used in RRMS may be mediating its effects at least in part through complement C3 inhibition. The use of DMF in EAE mice reduced iNOS + pro-inflammatory macrophages/microglia, decreased C3 deposition in the CNS and suppressed the development of reactive C3 + A1 astrocytes (Yadav et al., 2021). Reactive C3 + GFAP + astrocytes are highly neurotoxic and are involved in axonal loss and suppression of remyelination (Liddelow et al., 2017). DMF may reduce C3 synthesis in MS by suppressing proinflammatory cytokines’ production and TLR signaling pathways. Since C3 + reactive astrocytes are induced by IL-1α, TNFα, IL6, and C1q produced by activated reactive microglia, DMF treatment may also suppress the development of these C3-secreting reactive astrocytes by decreasing iNOS + proinflammatory microglia and macrophages (Yadav et al., 2021).

### C1q in experimental autoimmune encephalomyelitis: Important in cuprizone-induced experimental autoimmune encephalomyelitis, but not in myelin oligodendrocyte glycoprotein models

Evidence from animal models suggests that complement C1q, a complex glycoprotein, may also have an important role in MS pathogenesis. Elevated C1q levels were associated with an increased spinal cord lesion volume in the cuprizone-induced EAE mice model (Gao et al., 2022). In another study, knockdown of the C1s subunit of C1 in cuprizone-induced demyelination mice substantially reduced demyelination, modulated the microglia phenotype toward an anti-inflammatory one, and improved neurological function (Figure 3). The effects of the C1 complex in the white matter were presumed to be exerted *via* Wnt signaling (Gao et al., 2022). In these knockdown mice, β-catenin expression and its nuclear translocation in oligodendrocyte progenitor cells (OPCs) was inhibited. *In vitro*, C1q treatment increased the levels of LRP-6 ECD (a Wnt receptor) and β-catenin expression in OPCs, an effect that was reversed by C1s silencing. The inhibition of C1s also lowered the number of OPCs and enhanced the number of mature oligodendrocytes and MBP. This suggests that the C1 complex may be involved in demyelination in response to cuprizone in mice by preventing the differentiation of OPCs into mature oligodendrocytes through Wnt/β-cateninin signaling. Therefore, the inhibition of complement protein C1q or C1s in the early stages after an acute MS relapse may potentially help with remyelination efforts (Gao et al., 2022). C1q may also be a critical mediator of microglia activation, and, in MS, it can potentially modulate the switch of these cells to an inflammatory profile, also known as disease-associated microglia (DAM). Conditional C1q cKO in mice microglia eliminated C1q immunoreactivity in the CNS, suggesting that these cells are the primary source of this protein in the brain. After EAE induction in these C1qcKO mice, the density of Iba1 + cells and reactive microglia was significantly decreased (Absinta et al., 2021). Together, this data demonstrated a role of autocrine C1q in mediating microglia reactivity and driving inflammatory neurodegeneration. As mentioned above, C1q KO did not prevent MOG_35–55_-induced EAE in C57BL/6 mice (Figure 3). However, infusion of anti-MOG antibodies in these C1q KO EAE mice did not exacerbate disease severity when compared to WT mice, suggesting that the classical complement system may be the dominant effector cascade activated by demyelinating antibodies (Urich et al., 2006; Figure 3). This is significant as IgM and IgG antibodies have been found in about 50 and 75% of MS patients, respectively. These are present on the axons and oligodendrocytes of acute, chronic active and chronic inactive lesions as well as in NAWM (Sadaba et al., 2012). These immunoglobulins can cause complement-dependent antibody-mediated axonal loss in *in vitro* cultures (Elliott et al., 2012; Peschl et al., 2017). In one study, IgG1 myelin-specific MS recombinant antibodies from clonally expanded plasmablasts recovered from MS patients’ CSF triggered complement-dependent cytotoxicity, astrocyte activation and demyelination of mouse organotypic cerebellar slices (Liu et al., 2017) and spinal cord explant cultures (Blauth et al., 2015). Evidence from electron microscopy studies also showed the presence of anti-myelin antibody-complement complexes that are capable of opsonizing the myelin surface, making it a target for macrophages and microglia, resulting in myelin stripping (Romanelli et al., 2016). Interestingly, direct injection of anti-MOG antibodies with mouse complement in the corpus callosum of WT mice was sufficient to cause activated complement deposition and demyelination without the need for EAE induction (Berg et al., 2017).

All this evidence supports the importance of B-cell derived myelin antibodies in the pathology of MS, and of C1q and the classical complement cascade as central effectors of the pathogenic effects of these antibodies, likely through both complement-dependent cytotoxicity and increased opsonization of antibody-bound antigens. In addition, beyond its antibody-mediated effects, C1q may also act as a modulator of cellular responses in MS including microglia activation and the regulation of oligodendrocyte differentiation.

### Is C3 critical for experimental autoimmune encephalomyelitis pathogenesis? The role of scaveneger receptors

Although all the evidence described above points toward a role of C3 in EAE, one study on C3 KO MOG_35–55_-induced EAE mice found that C3 KO mice were equally susceptible to EAE as C3 + / + mice were, although the onset of EAE was delayed in C3 KO mice. In addition, no differences were found in the production of proinflammatory cytokines (i.e., IL-2, IL-4, IL-12, TNF, and IFNy) (Calida et al., 2001). In this study, it was suggested that C3 and the complement system may have a protective action against the detrimental effects of certain proinflammatory cytokines including TNF, as enhanced production of TNF and IL-1β, and increased susceptibility to endotoxin challenge has been observed in C3 KO mice (Fischer et al., 1997). It is important to note that the MOG_35–55_ mice model induces a primarily T cell mediated response which may not recapitulate all the scope of complement pathogenesis in human MS. However, given that these mice showed demyelination of the brain and spinal cord in the absence of complement activation, this implies that additional mechanisms may be acting as effectors of demyelination. It is well-known that myelin opsonized with anti-myelin antibodies or complement is taken up more efficiently by phagocytic microglia and macrophages. This complement-mediated opsonization of myelin and phagocytosis appears to be a contributing mechanism in the demyelination that occurs in MS plaques. However, blocking the complement in the absence of autoreactive antibodies does not fully prevent myelin uptake. When microglia and myelin were incubated with heated mouse serum, which inactivates all serum complement, phagocytosis was decreased but not abolished (Reichert and Rotshenker, 2003), indicating that other mediators and receptors may be involved (Hendrickx et al., 2013). In addition, myelin can directly bind to CR3 without the need for complement opsonization and promote proinflammatory cytokine secretion through the FAK/PI3K/Akt/NF-kb signaling pathway, thus serving as an endogenous inflammatory stimulus (Sun et al., 2010).

Scavenger receptors (SRs) are a diverse superfamily of cell surface receptors that can act as pattern-recognition receptors and recognize, phagocytose, and clear various pathogen associated molecular patterns (PAMPs) and oxidized epitopes. It has been shown that SR ligands on oxidized LDL and apoptotic cells are present in the myelin of MS lesions (Haider et al., 2011). Malondialdehyde, an oxidation-specific epitope is present in active MS lesions (Haider et al., 2011). Immunization with malondialdehyde-modified MOG aggravated EAE, and *in vitro* phagocytosis studies showed that this modified MOG was phagocytosed more efficiently by macrophages *via* the class A SR (SR-AI/AII) (Wallberg et al., 2007). In turn, blocking SR-AI/AI reduced the uptake of modified MOG (Wallberg et al., 2007) and myelin *in vitro* by mouse macrophages and microglia (da Costa et al., 1997). SR-A KO mice also showed a less severe EAE disease course with diminished demyelination (Levy-Barazany and Frenkel, 2012). Mice treated with neutralizing antibodies against another SR, CXCL16, also had a delayed EAE onset and less severe disease (Fukumoto et al., 2004). Finally, another SR, LRP-1, was shown to bind myelin directly *in vitro*, thus facilitating its uptake by rat microglia, astrocytes, and oligodendrocytes (Gaultier et al., 2009). In another study, CD68, CXCL16, SR-AI/AII, LOX-1, FcyRIII, and LRP-1 mRNA were upregulated in the rim of chronic active MS lesions. IHC also revealed CD68, CXCL16, and SR-AI/II expression by foamy macrophages and ramified microglia around chronic active MS lesions (Hendrickx et al., 2013). This data suggests a role of SRs in myelin uptake and phagocytosis in EAE, an event that is independent of complement and antibody-binding, and that could serve as an additional mechanism mediating demyelination in MS.

### Complement as an immune modulator

Complement proteins can also act as linkers between the innate immune and adaptive immune response in MS animal models. In this section we will review the current evidence favoring these immune modulatory roles of complement.

**FIGURE 4:**
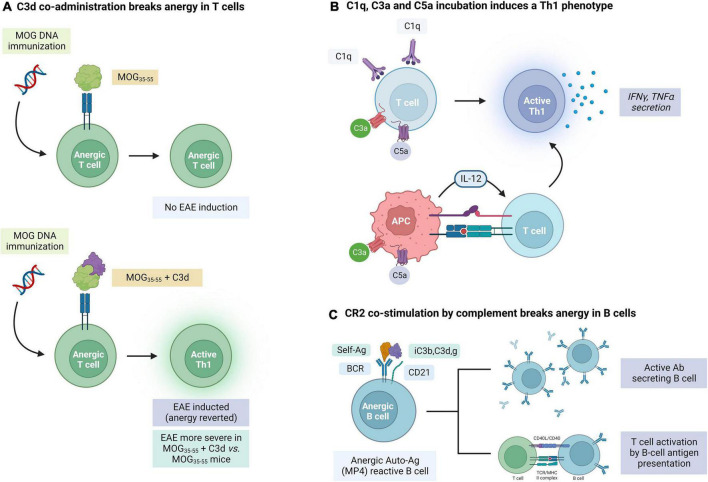
Evidence for the role of complement in the modulation of adaptive immune responses mediated by T and B cells in experimental autoimmune encephalomyelitis (EAE). **(A)** C3d co-administration breaks the anergy in MOG_35–55_ self-reactive T cells. **(B)** T cells can also be activated by C1q, C3a, and C5a through direct (T cell binding) and indirect (APC activation) mechanisms. **(C)** Concurrent binding of an antigen and complement factors iC3b, C3d, and C3g to complement receptor 2 (CR2) can break the anergy of self-reactive B cells. The figure was created with BioRender.com.

It is known that C3d may act as an adjuvant of the humoral B-cell mediated response upon binding to myelin antigens. During the induction phase of EAE in C57BL/6J mice, immunization with human MOG_35–55_ coupled to C3d was found to accelerate the appearance of clinical signs of disease and enhance the severity when compared with MOG_35–55_-immunized WT mice. This correlated with an increased infiltration of leukocytes in the CNS, increased complement activation and development of areas of demyelination and axonal loss. Interestingly, B cells in these mice had an increased capacity to act as antigen-presenting cells (APCs) and form germinal centers. In addition, the production of MOG-specific Abs was enhanced following dual MOG/C3d immunization. This suggests that C3d binding to self-antigens in autoimmune disease can enhance the adaptive immune response (Jégou et al., 2007b). In another study, infusion of two or three copies of C3d fused to MOG_35–55_ could break tolerance and induce EAE in mice that had been previously vaccinated with MOG-DNA, which has been proven to protect from EAE induction in WT mice (Jégou et al., 2007a; Figure 4A). These results indicate that C3d can revert T cell anergy induced by MOG-DNA by increasing MOG presentation to these cells and providing efficient costimulatory signals. C3 KO mice also had reduced numbers of CD4 and CD8 T cells, with lower IFNy, supporting the role of this factor in promoting T cell mediated responses (Szalai et al., 2007). In addition, C3d may also be able to enhance the uptake, processing, and presentation of opsonized antigens to B cells (Hess et al., 2000; Cherukuri et al., 2001), an effect that is likely mediated by binding of C3d to CR2 in these B cells. All this evidence suggests a role of C3d as an immune modulator beyond its activity as an opsonin.

In addition to C3d, other complement factors also have immune modulating properties. Apart from its effects on microglial activation, C1q can induce the activation of T cells and the secretion of IFNγ and TNFα by these cells (Chen et al., 1994). As it will be described in more detail below, chemokines C3a and C5a can also enhance inflammatory responses in T cells, promote their proliferation and diminish apoptosis (Kwan et al., 2012; Figure 4B). Complement may also affect the immune response by modulating APC maturation and cytokine production. Binding of C3a and C5a to their complement receptors on APCs leads to production of IL-12 which promotes Th1 immunity (Lalli et al., 2007; Li et al., 2008). C3aR and C5aR deficient mice were also found to have a weak response on T cell stimulation (Strainic et al., 2008).

Most of the studies on the effect of immune complement signaling in animal models have been done in the T-cell dependent but B-cell independent MOG_35–55_ EAE model, which has some limitations in assessing the full scope of complement involvement in EAE and MS pathogenesis. Using MBP-PLP (MP4)-immunized WT C57BL/6 mice that develop EAE through a B cell dependent mechanism, Hundgeburth et al. observed that demyelinated lesions in the CNS of these animals colocalized with C3/C3b and MAC deposition (Kuerten et al., 2006) in the spinal cord and cerebellum. When complement was depleted using CVF prior to immunization, incidence of disease decreased in these mice. These findings further support the role of complement activation in antibody-mediated demyelination and axonal injury (Elliott et al., 2012; Blauth et al., 2015; Romanelli et al., 2016; Liu et al., 2017; Peschl et al., 2017). More importantly, and in line with the role of complement as an immune modulator, T cell and B cell responses as assessed by MP-4 specific cytokine and antibody responses, were also significantly attenuated in these CVF-treated mice when compared to controls, while production of MP-4 specific IgG was also decreased (Kuerten et al., 2006).

This evidence suggests that complement signaling may also be involved in modulating B cell mediated responses in MS. CD21, also known as CR2 is a cell surface glycoprotein expressed in B cells which recognizes multiple ligands including complement products iC3b and C3d,g and is also the ligand for EBV (Molina et al., 1996). Studies on CR2 KO have confirmed the importance of this receptor in the humoral response to T-dependent and T-independent antigens, the generation of memory B cells, and B cell survival in germinal centers (Carter et al., 1988; Ahearn et al., 1996; Molina et al., 1996; Fischer et al., 1998; Barrington et al., 2005). In addition, co-engagement of an antigen to the BCR and complement C3/C3b to CR2 on B cells reduces the threshold for B-cell activation (Figure 4C). Targeting C3d-opsonized antigens to CR2 was also found to promote specific T cell activation through enhanced antigen presentation by both antigen-specific and non-specific B cells (Boackle et al., 1998).

Complement receptor 2 (CR2) can also facilitate antigen recognition and uptake by B cells either by cross linking of CR2 and BCR or through a BCR-independent receptor. In contrast to the antigen-specific BCR, CR2 can recognize any antigens coated with fragments of complement proteins (i.e., C3) or immune complexes. This process can therefore bypass antigen specificity in B cells (Nielsen et al., 2001). Interestingly, in a recent study it was found that B cells, irrespective of their specificity, could engage in the presentation of the MS self-antigen MBP in a complement-dependent manner. When circulating B cells from healthy donors were placed in a serum free medium, only 3–4% was capable of binding MBP and presenting its immunodominant peptide MBP_85–99_. However, in the presence of serum with complement factors, most B cells bound MBP and almost half presented MBP_85–99_. This process was dependent on the presence of active complement factors and a functional CR2 (Brimnes et al., 2014).

Stimulation of BCR by an auto-antigen alone cannot overcome the inhibitor mechanisms in place in autoreactive anergic cells. In contrast, the interaction between C3d-linked antigens and CR2 has also proven to be capable of breaking anergy in autoreactive B cells. In one study, MP4 coated with C3d, but not MP4 alone, was capable of overcoming this anergic state and stimulating the production of MP4-reactive B cells (Lyubchenko et al., 2007). This suggests that maintenance of self-antigen tolerance in B cells depends on a fine balance that can be decompensated with the activation of the complement system.

Taken together these findings support an important role of the immune complement system in triggering antibody dependent demyelination, antigen-specific T cell immunity, and modulating antigen-specific B cell responses in EAE and potentially MS (Werneburg et al., 2020).

### Complement in the gray matter and synaptic dysfunction in experimental autoimmune encephalomyelitis models

**FIGURE 5:**
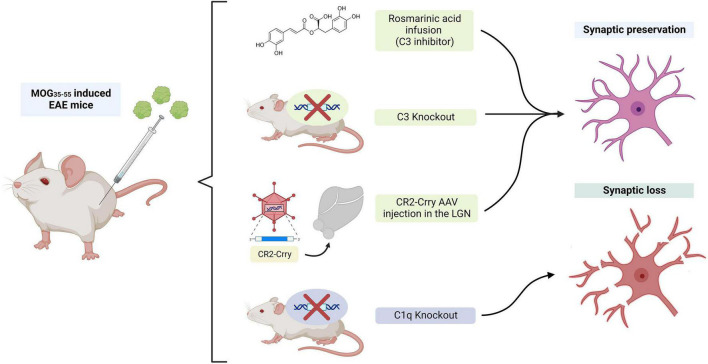
The role of C1q and C3 in synaptic pruning in experimental autoimmune encephalomyelitis (EAE) models. C3 inhibition resulted in synaptic preservation, while C1q KO did not have an effect in the prevention of synapse loss in these animals. The figure was created with BioRender.com.

Complement protein C3 may also be involved in the synaptic dysfunction and cognitive impairment that occurs in the gray matter and hippocampus of MS. In early stage MOG_35–55_-induced EAE mice, the expression of complement pathway genes was elevated in the dentate gyrus. C3 mRNA had a 10-fold upregulation in this region, while there was no increase of downstream factors such as the terminal component C5 (Bourel et al., 2021). RNAseq analysis suggested that C3 was synthesized by active microglia in this region. In addition, pharmacological inhibition of C3 activity by administration of rosmarinic acid in MOG_35–55_-induced EAE mice prevented memory impairment, microglia-mediated synapse phagocytosis and dendritic loss in the dentate gyrus (Figure 5). Similarly, when EAE was induced in C3 KO mice, dendrites and spines in the dentate gyrus were preserved, microglial activation was reduced (Hammond et al., 2020) and memory abilities remained intact (Bourel et al., 2021; Figure 5). In a different study, inhibition of NLRP3 inflammasome activation in EAE mice alleviated hippocampal pathology and synapse loss (Hou et al., 2020). This effect was presumed to be mediated by the prevention of astrocytes’ conversion to the A1 phenotype and the subsequent inhibition of C3 release by these cells. Interestingly, clinical evidence has also revealed that certain C3 polymorphisms correlated with worse cognitive performance, lower gray matter volume, lower brain parenchymal fraction and higher white matter lesion burden (Roostaei et al., 2019).

To further define the role of complement-mediated synapse loss and microglial reactivity Werneburg et al. (2020) developed a targeted approach to interfere with C3 activity. Lateral geniculate nucleus neurons of the thalamus were infected with an AAV viral vector containing the protein Crry, the membrane bound inhibitor of C3. After EAE induction, mice treated with the Crry viral vector had decreased microglial engulfment of presynaptic terminals, decreased synapse loss and improved visual function, reinforcing the role of complement C3 on microglial-mediated synaptic pruning in EAE and MS (Figure 5). These effects were presumed to be largely mediated by the interaction between complement fragments iC3b and C3b, and complement receptors CR3 (CD11b/CD18) (Schafer et al., 2012; Bajic et al., 2013) and CR1 (CD35), respectively, which are expressed in microglia with phagocytic properties.

C1q deposits were also found in the dorsal hippocampal CA2 pyramidal layer of cuprizone EAE mice, which also exhibited impaired encoding of social memories. These deposits colocalized with inhibitory synapses engulfed by microglia and macrophages (Ramaglia et al., 2021). However, C1qa KO EAE mice had little to no change in synapse loss and microglial activation in the hippocampus, EAE clinical score and memory/freezing behavior (Hammond et al., 2020; Figure 5). As mentioned above, beyond animal models, Michailidou et al. described C1q expression and C3 activation in the hippocampi of MS patients, with an associated decrease in synaptic density. These proteins also localized with HLA-positive cell processes and lysosomes, enforcing their role in the engulfment of tagged synapses by microglia (Michailidou et al., 2015).

In addition to synaptic pruning, the gray matter of patients with MS can also develop demyelination. Cortical lesions in MS patients are classified based on their location in cortico-subcortical, intracortical or subpial (Kidd et al., 1999; Peterson et al., 2001), and these cortical lesions may contribute to clinical symptoms and disease progression in chronic MS (Kutzelnigg et al., 2005). One limitation of studying cortical lesions in animal models is that rodent EAE models rarely have brain involvement. However, Merkler et al. (2006a) found that stereotactical injections of pro-inflammatory cytokines in the cortex of Lewis rats immunized with a subthreshold dose of MOG led to the formation of reversible cortical demyelination sharing similarities with human cortical MS. These animals exhibited transient inflammatory infiltrates, terminal complement deposition and demyelination in the gray matter. Terminal complement activation and MAC formation, as assessed by C9 deposition, was most pronounced shortly after cytokine injection during cortical inflammation and demyelination and resolved after. These rats also exhibited a high regenerative capacity following a single episode of inflammatory demyelination. This contrasted with the white matter changes seen in these animals where dense macrophage and activated microglia infiltrates persisted without significant remyelination. These findings also correlated with the relative lack of inflammation seen in chronic demyelinated cortical MS lesions of patients (Bø et al., 2003). This may suggest that the extensive and fast remyelination within the cerebral cortex may mask cortical demyelination in early multiple sclerosis, and the extensive cortical demyelination found in some late-stage chronic patients can represent an exhaustion of this remyelinating capacity.

### Complement in other symptoms of multiple sclerosis: Optic neuritis, emotional dysfunction, and neuropathic pain

The immune complement system may also be involved in the pathogenesis of retinal ganglion cell loss (RGC) associated with optic neuritis in MS. In EAE mice models, conditional deletion of C3 in astrocytes protected the retinal ganglion layer from neurodegeneration. This implies that astrocyte C3 expression may be a critical mediator of retinal neuronal pathology in EAE and MS (Gharagozloo et al., 2021). RNA sequencing analysis also showed enrichment of the complement cascade and expression of complement protein C3 in optic nerve astrocytes of EAE mice (Tassoni et al., 2019). At peak EAE, iNOS + microglia expressing C1q, proinflammatory cytokines TNFα and IL-1α, and A1 neurotoxic astrocytes expressing high levels of C3 are prominent in the optic nerve tissue of these mice (Jin et al., 2019). However, the retinal pathology with RGC reduction and postsynaptic protein and neurite compromise does not manifest until weeks later, suggesting a latency period between glial activation and complement activation, and neuronal injury in this model of EAE optic neuropathy. The evidence for the involvement of the immune complement in optic neuritis has gone beyond the studies on animal models. GWAS analysis of MS patients found that C3 gene variants were associated with OCT-assessed ganglion cell/inner plexiform layer atrophy in MS patients. C1QA and CR1 variants were also associated with sustained low-contrast letter acuity loss in these patients (Fitzgerald et al., 2019). This suggests that early complement pathway gene variants are associated with visual system degeneration in MS.

Emotional dysfunction is common in patients with multiple sclerosis (MS), and in EAE and the pathophysiology of this is poorly understood. In a recent study, the basolateral amygdala of EAE mice was evaluated in early EAE before demyelination, T-cell invasion and motor dysfunction. Interestingly, these mice had an increased frequency of excitatory post-synaptic potentials (EPSCs), and, in contrast to the above findings, in this disease stage microglia activation was downregulated and complement protein C3 expression was also reduced (Acharjee et al., 2018). This correlated with an increase in dendritic spine density and AMPA-NMDA ratio, indicating an increased number of new glutamatergic synapses due to decreased C3-mediated synaptic pruning by microglia. These results contrasted with what is seen in other brain regions in EAE, where there is microglia activation and significant complement deposition. One limitation of the study was that only early EAE was assessed. More research is thus needed to evaluate how synaptic dysfunction in the amygdala evolves as the disease progresses in EAE mice models.

The complement system may also be involved in the neuropathic pain of patients with MS. In a MOG_35–55_-induced EAE model, exam of lumbar dorsal root ganglia (DRG) neurons revealed upregulation of the mRNA of C3 and receptors C3aR1, C5aR1 at the onset of EAE, as well as a transient increase in C5aR1 + immune cells, CD4 + T-cells and Iba1 + macrophages. The DRG also had upregulation of mRNA transcripts of the NLRP3 inflammasome, IL-1β and IL-18 at disease onset. Electrophysiological analysis revealed hyperexcitability in medium-to-large fiber neurons, concluding that immune activation and injury to the DRG neurons, in part mediated by complement, can contribute to peripheral sensitization and neuropathic pain in MS (Yousuf et al., 2019).

### Complement and viral infections in multiple sclerosis

Mounting evidence has suggested the importance of EBV and HHV-6 infection in the pathogenesis of MS (Stuve et al., 2004; Virtanen et al., 2011; Tzartos et al., 2012; Meier et al., 2021; Bjornevik et al., 2022). As reviewed by Shinjyo et al. (2021) viral infections, including HIV, herpes viruses, measles, TMEV, WNV, Zika, among other infectious agents, can modulate the complement system in the CNS, and this interaction may be involved in neurodegeneration and dementia. Therefore, one can assume that complement activation may also be playing an important role in the pathogenic effects of viral infections in MS. In this context, the viral infection animal models of MS, such as the TMEV and MHV-induced demyelination models, serve as a perfect template to explore the role of viruses in the pathogenesis of this condition and the importance of complement system activation (Friedmann and Lorch, 1985; Katz-Levy et al., 1999; Mecha et al., 2013; Gilli et al., 2016a,b). In line with this, using the TMEV model, Linzey et al. (2022) found an increased expression of both classical and alternative complement pathway components. However, only the classical complement pathway activation as assessed by the level of C1q, C3, and C3aR1 expression, correlated with worse disease outcomes. These findings contrasted with that of the PLP139–151 relapsing EAE model also used in the study, where activation of the alternative pathway as assessed by the level of CFb expression was associated with worsened disease severity, while C1q expression correlated with an improved acute disease course. In both models, these effects were independent of MAC formation. C1q and C3 deposits were also observed in the spinal cord of these TMEV mice, with prominent C1q deposition in regions of demyelination and axonal damage. The TMEV model is characterized by an important B cell mediated immune response. Linzey et al. also demonstrated that an increased IgG1 expression correlated with increased C1q and with worse disease outcomes in this model. This again supports the hypothesis that the classical complement is a central mediator of the effect of self-reactive antibodies in MS (Urich et al., 2006), and also favors the role of this pathway in the pathogenic effects of viral infections in this disease. However, more research is needed to elucidate this relationship, and the use of the TMEV, MHV and other viral models of MS will be crucial to explore this.

## Profiling the dual role of terminal complement activation and membrane attack complex formation

All pathways of complement activation can ultimately lead to the downstream cleavage of C5 to generate C5a, an anaphylatoxin, and C5b, an opsonin. C5b can then combine with complement proteins C6-C9 to form the membrane attack complex (MAC). C5a and MAC have been found to be major effectors of neuroinflammation and neurodegeneration in multiple CNS disorders (Dalakas et al., 2020; Schartz and Tenner, 2020). With this evidence, MS animal models have been employed to further evaluate the involvement of the terminal complement components and MAC in this disease.

After inducing chronic relapsing EAE in Biozzi ABH mice, Ramaglia et al. assessed complement activation and expression at different stages of the disease. Using IHC, MAC deposition was detected in acute and progressive disease, as well as in relapses. However, although mRNA expression of C1q and C3 was increased throughout the disease course, expression of C9, part of the MAC, was only increased in the acute phase and was significantly reduced in all other stages. Interestingly, expression of complement regulators CD55, Crry, and CD59a was also reduced in these mice (Boos L. A. et al., 2005; Ramaglia et al., 2015).

The deposition of C9 within the CNS was also studied in EAE rat models including inflammatory EAE induced by passive transfer (tEAE) of MBP-specific T cells, antibody-mediated demyelinating EAE, and a chronic model induced by active immunization with guinea pig spinal cord tissue in adjuvant (Linington et al., 1989a). Two patterns were seen in these rats, one comprised of diffuse C9 staining of tissue adjacent to inflammatory lesions, and another consisting of granular fibrillar C9 deposits around inflamed vessels in areas of active demyelination. The last pattern, with extensive, but transient, perivascular and subpial granular deposits of C9 was more prominent in antibody mediated demyelinating EAE. A similar pattern of granular C9 deposition was seen in demyelinating lesions of rats with actively induced chronic EAE. C9/MAC deposition was also found in close apposition with demyelination areas in the spinal cord of MOG_35–55_-induced EAE in Lewis rats (Piddlesden et al., 1993).

**FIGURE 6:**
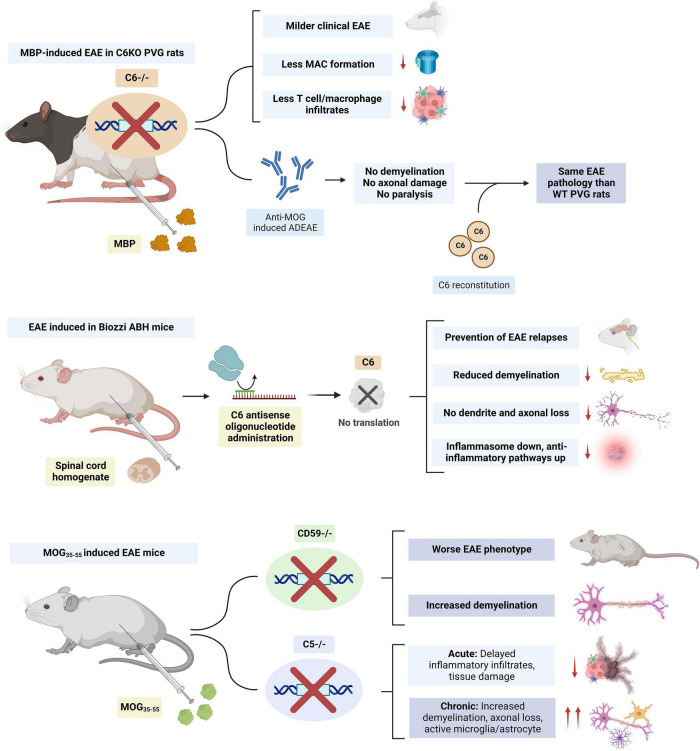
Summary of studies on the modulation of membrane attack complex (MAC) members (i.e., C5 and C6) in EAE. C6 KO had beneficial effects in both rat and mice models. Knockout (KO) of complement regulator CD59 resulted in a worse experimental autoimmune encephalomyelitis (EAE) phenotype and increased demyelination. C5 KO delayed inflammatory infiltrates in acute EAE but worsened the phenotype during the chronic stage. The figure was created with BioRender.com.

The course of active EAE was compared between PVG rats deficient in the C6 component of complement (PVG/C6-), unable to form MAC, and normal PVG rats. After immunization with MBP, C6KO rats developed a milder form of EAE, despite having a similar anti-MBP response and C3 deposition in the spinal cord (Tran et al., 2002; Figure 6). Less C9 was detected in the spinal cord of these rats consistent with the inability to form MAC. T cell and macrophage infiltrates were also significantly lower, which was presumed to be due to a reduced expression of P-selectin on endothelial cells, as this protein can be induced by the MAC (Mulligan et al., 1997). PVG/C6- rats also had lower peripheral WBC, neutrophil and basophil counts when compared to their littermates (Tran et al., 2002). Similar findings were observed in a different study where PVG/C6-rats immunized with MBP and given anti-MOG antibodies to induce Ab-mediated EAE failed to develop demyelination, axonal damage, or paralysis. In turn, when these animals were reconstituted with C6, they developed similar pathology and clinical disease to WT rats (Mead et al., 2002; Figure 6). Thus, in these models, complement activation, myelin opsonization by C3 and generation of anaphylatoxins C3a and C5a was insufficient to initiate demyelination without the presence of MAC.

Similar findings have been observed in mice models. In Biozzi AB/H EAE mice, subcutaneous administration of an antisense oligonucleotide against murine C6 mRNA prevented relapses, CNS demyelination, axonal, and synaptic loss. Inhibition of MAC formation led to downregulation of the NLRP3 inflammasome and upregulation of anti-inflammatory pathways (Michailidou et al., 2018; Figure 6). The evidence for the role of the terminal complement in MS also comes from animal models where terminal complement regulators were depleted. MOG_35–55_-induced EAE mice deficient in the regulator CD59a, had increased demyelination and a more severe phenotype (Mead et al., 2004; Figure 6).

All this evidence supports a role of MAC in exerting noxious effects during the acute stages of MS. However, animal models also suggest that, during chronic disease, MAC formation may have restorative effects. In C5KO EAE mice, delayed inflammatory infiltrates and tissue damage were observed in acute disease. However, these mice had increased demyelination, axonal loss, and glial activation during chronic disease (Weerth et al., 2003). MAC may promote the remyelination process by protecting oligodendrocytes from death in chronic disease (Cudrici et al., 2006b; Rus et al., 2006a,b; Tegla et al., 2009) and by inducing the apoptosis of inflammatory and degenerating cells (Niculescu et al., 2003), leading to a more efficient recovery from acute EAE (Bonetti et al., 1997; Pender and Rist, 2001). The effects of MAC in promoting oligodendrocyte survival are presumed to be due to an inhibition of mitochondrial apoptosis signaling (Soane et al., 1999, 2001), downregulation of the Fas-FasL pathway (Niculescu et al., 2004; Cudrici et al., 2006a) and activation of the cell cycle (Rus et al., 1996, 1997; Soane et al., 2001). The action of MAC on oligodendrocyte cell cycle activation and survival may be mediated by the activation of voltage-gated potassium K(v) channel 1.3 (K_*v*_ 1.3) (Tegla et al., 2011). Mature human oligodendrocytes also express CD59 on their surface, which can protect them from complement attack. Interestingly, rat oligodendrocytes lack CD59 expression and are sensitive to complement, which may limit the translation of the role of MAC in these animal models to humans (Zajicek et al., 1995). A study suggested that antibodies against these complement regulator proteins could be present in the sera of patients with MS (Pinter et al., 2000). This could explain MAC targeting of oligodendrocytes and demyelination in the acute stages of the disease. However, further research has not been able to replicate these findings (Miyaji et al., 2014). Interestingly, human oligodendrocytes also express low levels of C1 inhibitor (C1-INH) and membrane cofactor protein mRNAs, which could contribute to the selective vulnerability of these cells to attacks from early complement components and opsonization (Hosokawa et al., 2004).

## Assessing C3a and C5a signaling

C3a and C5a are small cleavage fragments released by complement activation, which also act as potent mediators of neuroinflammation (Peng et al., 2009). As the rest of complement activation products, their role has also been explored in animal models of MS. However, despite its potential pathogenic role in other neurodegenerative conditions such as AD (Fonseca et al., 2009; Hernandez et al., 2017; Litvinchuk et al., 2018) and neuroinflammatory diseases such as bacterial meningitis (Gasque et al., 1998), the role of these factors in animal models of EAE appears more controversial.

**FIGURE 7:**
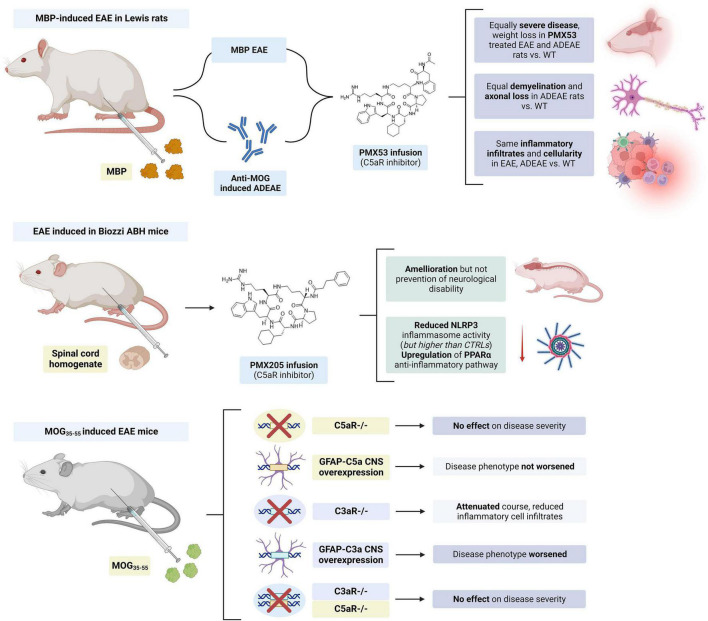
Summary of studies on the modulation of complement chemokines C3a and C5a in experimental autoimmune encephalomyelitis (EAE). PMX205 and PMX53 infusion had limited to no effect in disease severity. C5aR KO had no effect on the disease course and C5a CNS expression did not worsen the disease course in MOG_35–55_ mice. Meanwhile, C3aR KO improved and C3a CNS worsened EAE in these mice. However, concurrent KO of C3aR and C5aR had no effect on disease severity. The figure was created with BioRender.com.

The expression of C3a receptor (C3aR) on microglia and infiltrating macrophages was increased during EAE, while neuronal expression was unchanged (Davoust et al., 1999a). Similarly, the expression of the C5a receptor (C5aR) was also upregulated on microglia and hypertrophic astrocytes in the spinal cord of EAE Lewis rats (Nataf et al., 1998). However, the inhibition of C5aR in animal models with small molecule inhibitors (i.e., PMX205 and PMX53) (Morgan et al., 2004; Michailidou et al., 2018) as well as the knockout of this receptor (Reiman et al., 2002) had limited to no effects in influencing EAE disease severity. Selective expression of C5a in the brain also failed to exacerbate EAE in mice (Reiman et al., 2005). In contrast, C3aR deletion in MOG_35–55_-induced EAE mice attenuated the clinical course and reduced infiltration of inflammatory cells, while selective C3a CNS expression exacerbated this condition (Boos et al., 2004). However, when a double C3aR/C5aR KO EAE mice model was generated, a delayed onset but a similar course of disease was seen (Ramos et al., 2009; Figure 7).

Although the inhibition of their specific complement receptors did not significantly influence disease outcome, animal models suggest that C3a and C5a chemokines may modulate the interaction between APCs and T cells and promote T cell survival in EAE (Figure 4B). It has been described that the APC:T cell interaction produces C3a and C5a, and these factors may in turn be involved in the activation and generation of MOG_35–55_ specific IFNy and IL-17 producing T cells in EAE (Strainic et al., 2008). These effects are thought to be mediated by an increased IL-12 and IL-23 production (Strainic et al., 2008). These chemokines also increase T cell survival by upregulation of Bcl-2 expression and inhibition of Fas (Lalli et al., 2008). Other studies have also found that T cell activation can lead to autocrine complement activation and C3a/C5a synthesis. Upon TCR binding, there is intracellular generation of C5a and cleavage of C3 to C3a and C3b in these T cells. This intracellular C5a can activate C5aR1 leading to proinflammatory effects including the activation of the NLRP3 inflammasome (Arbore et al., 2016). Complement C3aR is present in both B and T cells and translocates to the plasma membrane upon T cell activation. Autocrine activation of C3aR by C3a in T cells resulted in increased Th1 effector functions, IFNy secretion and Notch signaling, as well as increased expression of mTOR and the NLRP3 inflammasome (Liszewski et al., 2013; Killick et al., 2018).

In one study the use of FUT-175, an inhibitor of C3/C5 convertase formation, was tested *in vitro* and in MOG_35–55_ induced EAE mice (Li et al., 2009b). *In vitro*, incubation with FUT-175 inhibited local C3a and C5a production by APC-T cell complexes and attenuated MOG_35–55_ specific Th1 and Th17 responses. *In vivo* administration of FUT-175 delayed EAE onset, lowered clinical scores, decreased CNS inflammation, and reduced demyelination. These mice also had decreased numbers of MOG_35–55_ specific IFNy and IL-17 producing T cells. Similar results were observed when adoptively transferred splenocytes from MOG_35–55_ immunized mice were transfected into naïve mice treated with FUT-175. Therefore, in contrast to prior studies, these findings suggested that the use of a complement regulator that modulates C5a/C3a production could have therapeutic efficacy in MS and EAE.

Decay-accelerating factor (DAF) is a membrane protein that inhibits cell surface complement activation. Absence of DAF lowers the restraint on junctional C3/C5 convertases formation which can result in increased levels of local C3a and C5a production (Liu et al., 2008). DAF-deficient APCs produced significantly more IL-12, C5a and IFNγ, which in turn stimulated T cells toward an IFNγ producing phenotype. Interestingly, this response was prevented by either C3a or C5a receptor deficiency in these APCs (Lalli et al., 2007). In line with the above findings, Daf1 KO mice displayed exacerbated disease progression and pathology in MOG_35–55_ EAE mice (Liu et al., 2005). When Daf1 transgenic mice with increased surface expression of DAF were generated, a delayed disease onset and lower clinical scores were observed. These mice also showed less inflammation and demyelination in their spinal cords and decreased MOG_35–55_ specific Th1 and Th17 cell responses. When co-cultured with CD4 + T cells and OVA_323–339_ peptide, DAF-overexpressing mouse dendritic cells produced less C3a and C5a and generated a milder T cell response (Li et al., 2009a).

Overall, these studies found that interfering with C3a and C5a synthesis through C3/C5 convertase inhibition could have a beneficial effect in EAE models. It needs to be argued, however, that interfering with C3/C5 convertase activity also affects the synthesis of C3b, C5b, and MAC formation. Therefore, the improved phenotype of these EAE models may be due to an interference on the synthesis of these other complement components rather than due to an effect on C3a and C5a production.

Adding to their complex interplay in EAE pathogenesis, it also appears that anaphylatoxins C3a and C5a may have role in neuroprotection. This was reviewed by Ingram et al. (2009). Pretreatment of primary murine corticohippocampal neurons with human or mouse recombinant C5a reduced glutamate neurotoxicity and neuronal apoptosis through MAPK-mediated regulation of caspase activation (Mukherjee and Pasinetti, 2001). C5aR-deficient mice were also more susceptible to apoptotic injury *in vivo* due to increased excitotoxicity (Mukherjee et al., 2008). Similarly, C3a was also found to be neuroprotective against glutamate toxicity in the presence of astrocytes (van Beek et al., 2001). In another study, selective expression of C3a in the CNS in C3a/GFAP mice resulted in more resistance to LPS endotoxin-induced lethality than WT and C3aR KO mice. Surprisingly C3a/GFAPxC3aR KO hybrids were also protected. This suggests that beyond the known C3aR, C3a may be exerting its protective effects through unidentified non-canonical pathways (Boos L. et al., 2005).

## Concluding remarks

In this review we described the use of animal models of MS to investigate the role of complement in the pathology of MS. The study of the role of complement in animal models of EAE has been ongoing since the 1980s with the initial use of CVF as a complement depleting agent in EAE Lewis rats (Abrahamson, 1971; Pabst et al., 1971; Linington et al., 1989b). From those initial studies, that set the path for the development of targeted therapeutics against the immune complement system, until today, great advances have been made to gain a better understanding of the pathogenic role of the different complement factors in MS. It appears that C3 and the alternative complement pathway may play a significant role in the demyelination mediated by opsonization and phagocytosis (Nataf et al., 2000; Hu et al., 2012, 2013; Ramaglia et al., 2012), as well as in the loss of synapses (Hammond et al., 2020; Hou et al., 2020; Werneburg et al., 2020; Bourel et al., 2021) that occurs in the acute and chronic phases of EAE. On the other hand, C1q and the classical complement pathway seem to be more important in the modulation of microglia activation (Absinta et al., 2021) as well as in antibody-mediated pathogenesis (Urich et al., 2006), as evidenced by the effects of C1q in EAE models of antibody-induced demyelination, in contrast to its more limited effect on MOG_35–55_ induced EAE. Complement activation products, and especially, anaphylatoxins C3a and C5a, may also serve as a bridge between the innate and adaptive immune response, by mediating both B and T cell activation (Lalli et al., 2007; Lyubchenko et al., 2007; Li et al., 2008; Strainic et al., 2008; Kwan et al., 2012). Beyond all these functions, the complement system may also have protective effects; for example, MAC formation may play a role in the regeneration of myelin after an acute attack and in promoting the survival of oligodendrocytes (Rus et al., 1996, 1997; Bonetti et al., 1997; Soane et al., 1999, 2001; Pender and Rist, 2001; Niculescu et al., 2004; Cudrici et al., 2006a); C3a and C5a in turn may also help in preventing glutamate excitotoxicity (Mukherjee and Pasinetti, 2001; Mukherjee et al., 2008; Ingram et al., 2009).

Overall, the action at multiple different levels of complement factors in animal models of EAE, including phagocytosis, immune cell response, chemoattraction and cytokine production, makes them a very attractive target for developing therapeutic interventions. In this review we have seen how these animal models can serve as a template for the development of new inhibitors. As an example, we have discussed the use of complement depleting agent cobra venom factor (CVF), the C3 inhibitor Crry, anti-factor B, the C3/C5 convertase inhibitor FUT-175, and the C5aR small molecule inhibitors PMX53 and PMX205. All these agents have been proven able to modify complement activity in these models and potentially affect the disease course (Morgan et al., 2004; Li et al., 2009b; Terenyi et al., 2009; Michailidou et al., 2018; Werneburg et al., 2020). It is for this reason that animal models serve as an ideal platform for the study of inhibitors targeting complement activation. As of today, no human clinical trial on the use of complement inhibitors for MS has been completed or is currently ongoing. However, we believe that MS animal models will aid in developing new complement modulators that could eventually translate for use in clinical trials.

Another critical challenge toward that goal is the development of better models that can mimic the course and pathogenesis of progressive MS, where therapeutic interventions are very limited. The use of progressive MS animal models would allow a better understanding of the role of complement in this specific context and thus potentially advance the use of compounds that affect this MS phenotype. It has been shown that immunization of aged Biozzi ABH mice with spinal cord homogenate (SCH) can lead to a monophasic persistent disease course that, clinically, may resemble primary progressive MS (Peferoen et al., 2016). The viral TMEV-induced demyelination model described above, also mimics some of the traits of progressive MS, including a progressive loss of functions, with predominance of innate immune mechanisms (Gilli et al., 2016a,b). However, the underlying neuropathogenesis in these mice needs to be further characterized to determine whether they can serve as a template for the study of progressive MS.

We hope that with the regained interest in the role of complement in this and other pathologies, the development of complement therapeutics with the aid of improved animal models of MS will see significant progress in the years to come.

## Author contributions

NS-C and OS contributed to the conception of this review. NS-C wrote the first draft of the manuscript. AB and OS contributed to manuscript revision and read. OS approved the submitted version. All authors contributed to the article and approved the submitted version.
